# Fenofibrate prevents iron induced activation of canonical Wnt/β-catenin and oxidative stress signaling in the retina

**DOI:** 10.1038/s41514-020-00050-7

**Published:** 2020-10-30

**Authors:** Ashok Mandala, Austin Armstrong, Becky Girresch, Jiyao Zhu, Aruna Chilakala, Sanmathi Chavalmane, Kapil Chaudhary, Pratim Biswas, Judith Ogilvie, Jaya P. Gnana-Prakasam

**Affiliations:** 1 Department of Ophthalmology, Saint Louis University, St. Louis, Missouri USA; 2 Department of Biology, Saint Louis University, St. Louis, Missouri USA; 3 Department of Energy, Environmental and Chemical Engineering, Washington University, St. Louis, Missouri USA; 4 Department of Medicine, Washington University, St. Louis, Missouri USA; 5 Department of Biochemistry and Molecular Biology, Saint Louis University, St. Louis, Missouri USA

**Keywords:** Retinal diseases, Pathogenesis

## Abstract

Accumulating evidence strongly implicates iron in the pathogenesis of aging and disease. Iron levels have been found to increase with age in both the human and mouse retinas. We and others have shown that retinal diseases such as age-related macular degeneration and diabetic retinopathy are associated with disrupted iron homeostasis, resulting in retinal iron accumulation. In addition, hereditary disorders due to mutation in one of the iron regulatory genes lead to age dependent retinal iron overload and degeneration. However, our knowledge on whether iron toxicity contributes to the retinopathy is limited. Recently, we reported that iron accumulation is associated with the upregulation of retinal and renal renin–angiotensin system (RAS). Evidences indicate that multiple genes/components of the RAS are targets of Wnt/β-catenin signaling. Interestingly, aberrant activation of Wnt/β-catenin signaling is observed in several degenerative diseases. In the present study, we explored whether iron accumulation regulates canonical Wnt signaling in the retina. We found that in vitro and in vivo iron treatment resulted in the upregulation of Wnt/β-catenin signaling and its downstream target genes including renin–angiotensin system in the retina. We confirmed further that iron activates canonical Wnt signaling in the retina using TOPFlash T-cell factor/lymphoid enhancer factor promoter assay and Axin2-LacZ reporter mouse. The presence of an iron chelator or an antioxidant reversed the iron-mediated upregulation of Wnt/β-catenin signaling in retinal pigment epithelial (RPE) cells. In addition, treatment of RPE cells with peroxisome proliferator-activated receptor (PPAR) α-agonist fenofibrate prevented iron-induced activation of oxidative stress and Wnt/β-catenin signaling by chelating the iron. The role of fenofibrate, an FDA-approved drug for hyperlipidemia, as an iron chelator has potentially significant therapeutic impact on iron associated degenerative diseases.

## Introduction

Iron plays a vital role in the retina with many iron containing proteins involved in the phototransduction cascade^1^. A stringent balance during iron uptake, transport, storage and utilization is required to maintain iron homeostasis^2^. However, excess iron promotes the generation of reactive oxygen species (ROS) through Fenton reaction causing molecular and cellular dysfunctions^3^. Age-related increases in iron levels have been found in the human and mouse retinas^4,5^. In addition, disrupted iron homeostasis in hereditary and acquired diseases such as aceruloplasminemia^6^, hemochromatosis^7,8^, Friedreich’s ataxia^9^, diabetic retinopathy (DR)^10^, age-related macular degeneration (AMD)^11^, glaucoma^12^, ocular siderosis^13^, multiple blood transfusions^14^, and excess dietary iron supplementation^15^ have been reported to cause retinal iron accumulation and degeneration.

Our recent study showed that retinal iron accumulation exacerbates cell death through oxidative stress, inflammasome activation, and enhanced renin–angiotensin system (RAS) activity^10^. RAS plays an important role in regulating vasoconstriction and electrolyte balance. Angiotensin II (Ang II) is the primary molecule of RAS produced as a result of cleavage of angiotensinogen (AGT) by renin and angiotensin-converting enzyme (ACE) sequentially. Ang II then binds to Ang II type 1 (AT_1_) and Ang II type 2 (AT_2_) receptors wielding diverse pathophysiological effects. Activation of retinal RAS due to concurrent upregulation of multiple RAS genes is associated with many ocular diseases including DR, AMD, uveitis, and glaucoma^16^. Recent studies established that all components of the RAS, including AGT, renin, ACE, AT1, and AT_2_ are downstream targets of Wingless-related integration site (Wnt)/β-catenin signaling^17^. However, it is not known whether iron can regulate retinal Wnt/β-catenin signaling. The Wnt signaling is a group of signal transduction pathways with diverse role during embryonic growth and development. In the canonical Wnt pathway, secreted Wnt ligands bind to the Wnt receptor Frizzled (Fzd) and co-receptor low-density lipoprotein receptor-related protein (LRP5/6) leading to its phosphorylation and activation^18^. This leads to inactivation of the “destruction complex” consisting of GSK-3β (glycogen synthase kinase-3β), Axin, and APC (adenomatous polyposis coli). This prevents the proteasomal degradation of β-catenin and promotes its accumulation and nuclear translocation. In the nucleus, β-catenin associates with T-cell factor (TCF) and regulates the expression of many target genes, including *VEGF* (vascular endothelial growth factor). Altered Wnt signaling has been reported as a contributing factor in various ocular disorders and malignancies^19–21^. Wnt activation may contribute to the angiogenesis, inflammation, and fibrosis^22^. In addition, the Wnt pathway affects cell adhesion through the binding of β-catenin and E-cadherin^23^. Wnt signaling has antagonistic pleiotropic effects, because they are quintessential earlier in life but deleterious later in life^19^. Downregulation of Wnt/β-catenin signaling and RAS blockade have been shown to protect against aging^24^. However, current therapy with RAS inhibitors have limited efficacy because of compensatory upregulation of renin expression^25^ necessitating the need for a new strategy to simultaneously target multiple RAS genes for a more effective treatment.

Peroxisome proliferator-activated receptors (PPARs) are nuclear receptors that play an important role as transcription factors in regulating the expression of genes involved in the lipid metabolism^26^. PPARα, PPARβ/δ, and PPARγ are the three isoforms, of which PPARα is crucial in the regulation of fatty acid oxidation, inflammation and angiogenesis^26^. Fenofibrate, a PPARα agonist, has been found to be effective in the amelioration of microvascular complications of diabetes in the FIELD and ACCORD trials^27–29^. Also, fenofibrate has been shown to potently block the activation of Wnt signaling in the kidney by destabilizing LRP6 mediated through inhibiting the renal ROS production^30^. In the present study, we determined if iron accumulation regulates canonical Wnt signaling in the retina and the therapeutic role of PPARα agonist fenofibrate in preventing iron-induced Wnt signaling.

## Results

### Iron induces canonical Wnt/β-catenin signaling in the retinal pigment epithelial cells

**Fig. 1:**
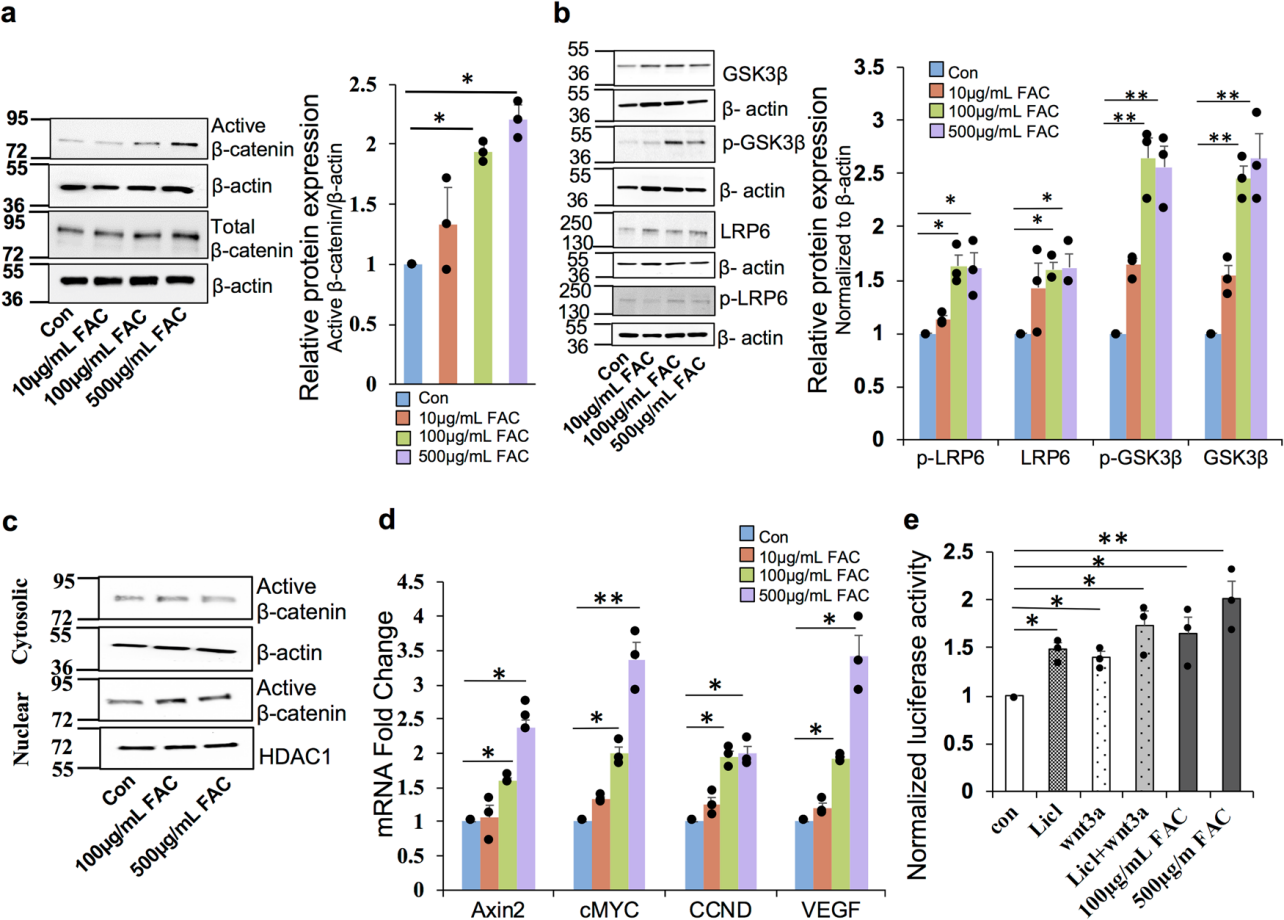
Iron induces canonical Wnt signaling in the retinal pigment epithelial cells. **a** Total and active β-catenin (non-phospho) protein levels were estimated by western blot in ARPE19 cells treated with different concentrations of FAC for 24 h. β-Actin was used as a loading control. **b** Protein expression of GSK-3β, p-GSK-3β, LRP6, and p-LRP6 were determined by western blotting. β-Actin was used as a loading control. **c** Expression of active β-catenin in cytosolic and nuclear fractions isolated from ARPE19 cells treated with FAC for 24 h. β-Actin and HDAC1 were used as loading controls for cytosolic and nuclear fractions respectively. In **a**–**c**, blots cropped from different parts of the same gel or from different gels are separated by white space. **d** mRNA expression of Wnt downstream target genes Axin2, cMYC, CCND, and VEGF in the cells treated with different concentrations of FAC for 24 h. **e** *Renilla luciferase* activity in the TCF/LEF Reporter transfected cells. LiCl and Wnt3a were used as a positive control. Data presented as mean ± SE of three independent experiments; *p < 0.05; **p < 0.001.

To investigate whether retinal iron accumulation during aging and degenerative diseases modulates canonical Wnt/β-catenin signaling, a human retinal pigment epithelial (RPE) cell line ARPE19 was treated with different concentrations of ferric ammonium citrate (FAC) for 24 h. FAC treatment increased the expression of total and non-phospho (active) β-catenin levels in a dose dependent manner (Fig. 1a). The expression levels of p-GSK-3β (Serine 9), GSK-3β, and LRP6 were upregulated by FAC (Fig. 1b). Further, FAC treatment increased the nuclear translocation of β-catenin as the active β-catenin levels in the nuclear fraction increased with FAC treatment but there was no change in the expression levels in the cytosolic fraction (Fig. 1c). mRNA expression of β-catenin downstream target genes *Axin2, cMYC, CCND* (cyclin D), and *VEGF* were significantly upregulated in FAC-treated human RPE cells in a dose dependent manner (Fig. 1d). Similarly, ARPE19 cells transfected with TopFlash reporter, increased the luciferase reporter activity in FAC-treated cells compared to the untreated cells (Fig. 1e). These results indicate that retinal iron overload inhibits GSK-3β and upregulates LRP6, thereby activating β-catenin signaling in the human RPE cells.

### Iron activates retinal canonical Wnt/β-catenin signaling in vivo

**Fig. 2:**
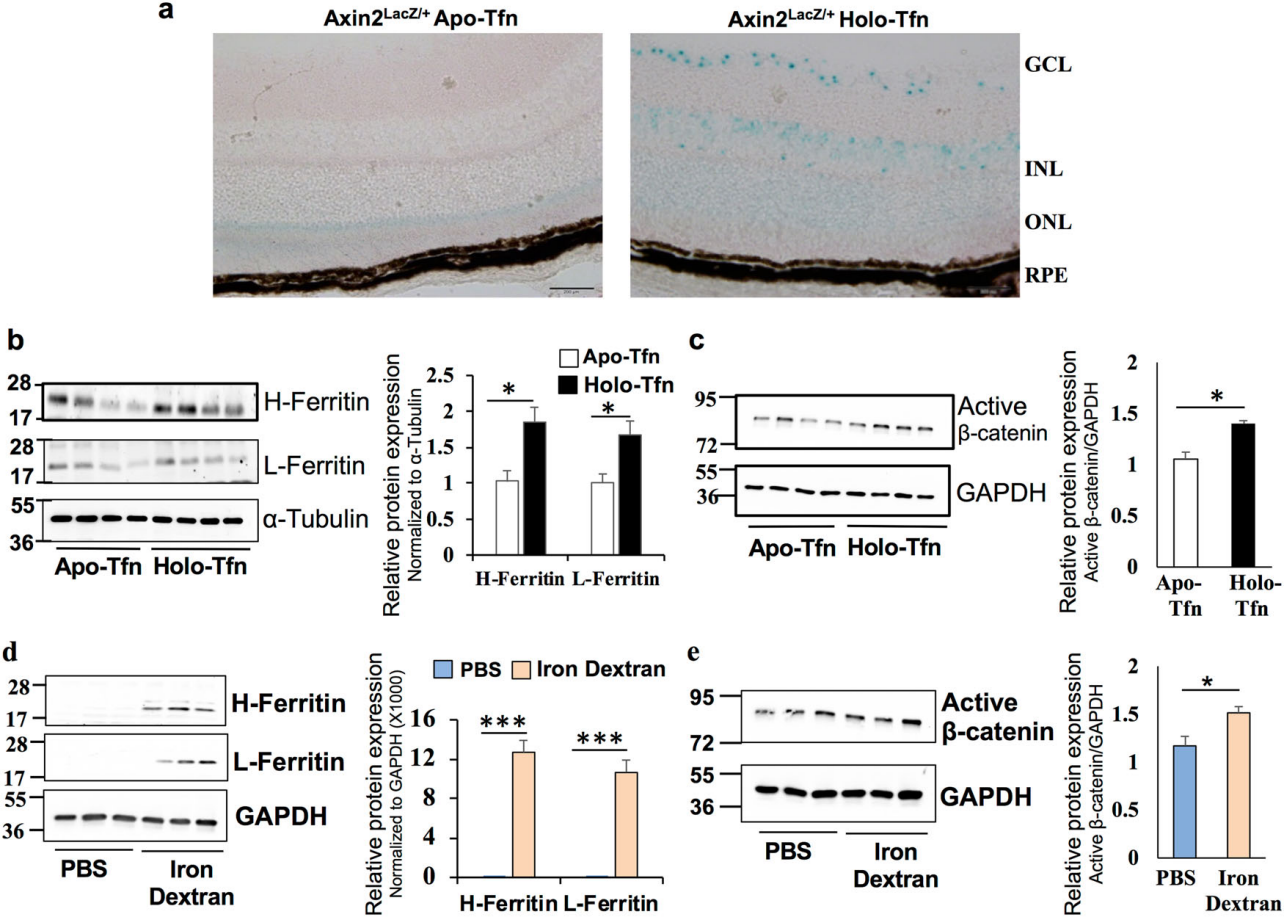
Iron activates retinal canonical Wnt/β-catenin signaling in vivo. **a** Representative images of retinal sections from Axin2^LacZ/+^ mice treated with apo- and holo-transferrin (Tfn), and stained for X-gal. GCL, ganglion cell layer; INL, inner nuclear layer; ONL, outer nuclear layer; RPE, retinal pigment epithelium. Heavy (H-) chain ferritin, light (L-) chain ferritin and active β-catenin protein levels were determined by immunoblotting after (**b**, **c**) intravitreal injection of apo- and holo-transferrin (Tfn) in the mice and (**d**, **e**) intraperitoneal injection of iron dextran for 8 weeks. α-Tubulin or GAPDH was used as an internal control. In **b**–**e**, blots cropped from different parts of the same gel or from different gels are separated by white space. Data presented as mean ± SE; *n* = 4–8 mice per group; *p < 0.05; ***p < 0.0001.

A Wnt signaling reporter mouse *Axin2*^*lacZ* /+^ was used to confirm if iron regulates retinal Wnt signaling in vivo. The *Axin2* gene is a known target of the canonical Wnt signaling and *Axin2*^*LacZ*^ mice were generated by a stable knock in of LacZ in frame with the start codon of the endogenous Axin2 to visually localize the Wnt signaling activation^31,32^. X-Gal (5-bromo-4-chloro-3-indolyl-β-d-galactopyranoside) staining of retinal sections from intravitreal holo-transferrin (iron)-treated eyes of *Axin2*^*lacZ*/+^ mouse showed upregulated Wnt signaling activity, mainly in the ganglion cell layer, inner nuclear layer, and, to a lesser extent, in the outer nuclear layer and RPE compared to the apo-transferrin (control)-treated eyes (Fig. 2a). To determine whether iron alters β-catenin protein expression in vivo, wild-type (WT) mice were administered with holo-transferrin (iron) or apo-transferrin (control) intravitreally and the retinal β-catenin levels were compared. We first confirmed that holo-transferrin treated retina had iron accumulation by checking the protein levels of H- (heavy chain) and L- (light chain) ferritin, an indirect indicator of intracellular iron levels (Fig. 2b). Holo-transferrin treated retina exhibited increased expression of active β-catenin compared to the apo-transferrin treated retina (Fig. 2c). To check if systemic iron overload can cross the blood-retinal barrier and regulate retinal Wnt signaling, mice were treated with iron-dextran intraperitoneally. The expression of H- and L-ferritin was higher in the retina of mice treated with iron dextran than the phosphate-buffered saline (PBS)-treated group (Fig. 2d). Further, iron dextran treated mice showed upregulation of active β-catenin in the retina (Fig. 2e). These results imply that both systemic and localized iron overload activates canonical Wnt/β-catenin signaling in the retina.

### Iron accumulation and enhanced Wnt signaling in the rd1 mouse model of Retinitis Pigmentosa

**Fig. 3:**
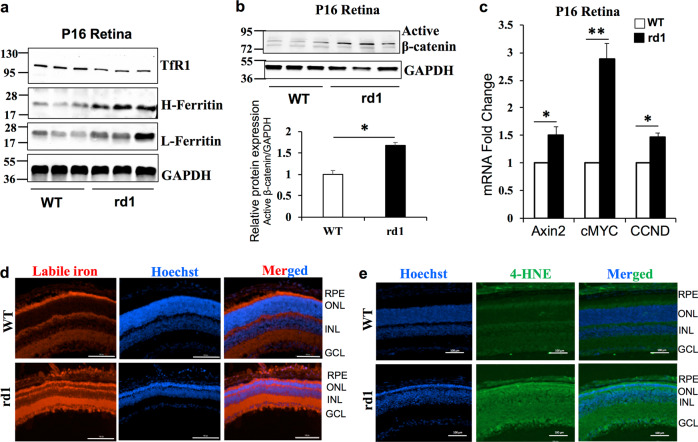
Iron accumulation and enhanced Wnt signaling in the rd1 mouse model of Retinitis Pigmentosa. **a** TfR1, Heavy (H-) chain ferritin, light (L-) chain ferritin, and **b** active β-catenin protein levels were determined by immunoblotting in WT and rd1 mouse at postnatal day 16. Blots cropped from different parts of the same gel or from different gels are separated by white space in **a** and **b**. **c** mRNA expression of Wnt downstream target genes Axin2, cMYC, CCND in WT, and rd1 mice retina at P16. **d** Representative images of retinal sections from P16 WT and rd1 mice stained for labile iron using FeRhoNox-1 fluorescent imaging probe. **e** 4-Hydroxynonenal staining in WT and rd1 mouse retina at P16. Scale bar is 100 μm for all the panels in **d** and **e**. Data presented as mean ± SE of three independent experiments; *n* = 3 mice per group; *p < 0.05; **p < 0.001.

To check whether iron accumulation is associated with increase in Wnt signaling during pathological conditions, we used rd1 mouse model of Retinitis Pigmentosa (RP). RP is the leading cause of inherited retinal blindness in the United States and is characterized by progressive degeneration of rod photoreceptors with no effective treatments currently available^33^. Oxidative stress has been shown to play an important role and the use of antioxidants reduced rod cell death in the retinal degeneration rd1 and rd10 mouse models of RP^33^. Importantly, the zinc–desferrioxamine complex has been shown to attenuate retinal degeneration in rd10 mouse model of RP by chelating the labile iron^34^. However, there is no literature on the iron status in rd1 or rd10 mouse models of RP. Here we show that H- and L-ferritin levels are higher in the rd1 mouse retina at postnatal P16 by which time more than half of the rod photoreceptors have degenerated (Fig. 3a). Binding of iron regulatory proteins to iron response element in the 3′-untranslated region of TfR1 regulates the expression of TfR1 resulting in TfR1 levels to be inversely proportional to the cellular iron status^2^. Thus, a decrease in TfR1 levels indicates iron accumulation in the retinas of rd1 mice (Fig. 3a). Similarly, active β-catenin and downstream target genes were significantly upregulated in the retinas of rd1 mice compared to WT mice at P16 (Fig. 3b, c). Labile iron staining using FeRhoNox-1 fluorescent imaging probe confirmed increase in Fe^2+^ iron accumulation in not only the RPE and outer nuclear layer containing the photoreceptors but also distributed throughout the nuclear and plexiform layers of the rd1 mice retina at P16 (Fig. 3d). 4-hydroxynonenal (4-HNE), a lipid peroxidation product, is upregulated during iron-mediated oxidative stress^35^. Similar to the labile iron staining, retinal sections stained for 4-HNE revealed robust increase throughout the rd1 retinas compared to WT retinas at P16 (Fig. 3e). In postnatal P6 and P10 mice, we found a similar increase in ferritin and active β-catenin protein levels in the rd1 mice retinas compared to WT retinas (Supplementary Fig. 1a, b), indicating that rd1 mouse model of RP has iron accumulation and associated increase in retinal Wnt signaling even before the photoreceptor degeneration starts at P10, thereby implying a contributory role for iron in the progression of RP.

### Iron-induced activation of Wnt signaling is mediated by oxidative stress

**Fig. 4:**
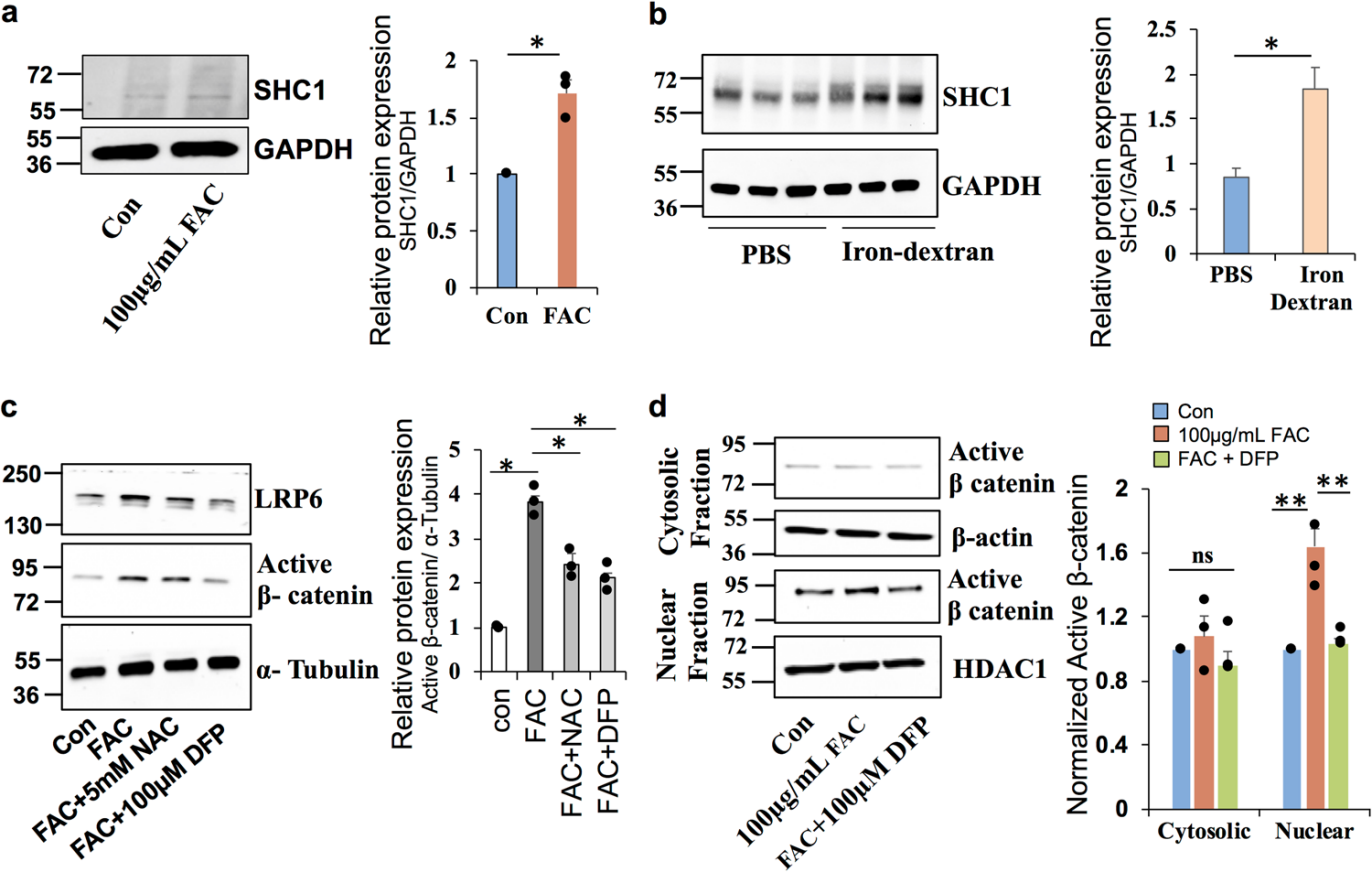
Iron induced Wnt signaling activation is mediated by oxidative stress. **a**, **b** Expression of Src collagen homolog (SHC1), an indicator of redox imbalance and oxidative stress, in the ARPE19 cells treated with FAC for 24 h and in mice treated with intraperitoneal iron dextran for 8 weeks. **c** ARPE19 cells when treated with FAC for 24 h along with antioxidant N-acetyl cysteine (NAC) or iron chelator deferiprone (DFP) normalized active β-catenin and LRP6 levels in western blotting. **d** Cytosolic and nuclear fractions were separated and the expression of active β-catenin was determined by western blotting. β-Actin and HDAC1 were used as loading controls for cytosolic and nuclear fractions respectively. Blots cropped from different parts of the same gel or from different gels are separated by white space. Data presented as mean ± SE of three independent experiments. *p < 0.05; **p < 0.001.

Oxidative stress has been reported to play an important role in the Wnt3a ligand-induced canonical Wnt/β-catenin signaling in endothelial cells^36^. RPE cells treated with FAC in vitro and mice treated with iron dextran in vivo upregulated the expression of Src collagen homolog (p66 Shc1) (Fig. 4a, b), an indicator of redox imbalance that plays a vital role in Wnt3a-induced oxidative stress and canonical Wnt signaling activation^36,37^. To investigate whether the iron-mediated oxidative stress is responsible for the activation of Wnt signaling in the retina, cells were treated with antioxidant *N*-acetyl cysteine (NAC, 5 mM) or iron chelator deferiprone (DFP, 100 µM) in the presence of FAC. Treatment with NAC or DFP significantly reduced the FAC-induced upregulation of LRP6 and active β-catenin (Fig. 4c). Further, western blotting in the nuclear and cytosolic fraction demonstrates that DFP treatment significantly reduced the translocation of active β-catenin to the nucleus (Fig. 4d). In summary, these results establish that iron induces ROS generation and increases LRP6, activating the Wnt signaling in the retina.

### PPARα agonist fenofibrate prevents iron-induced Wnt signaling in ARPE19 cells

**Fig. 5:**
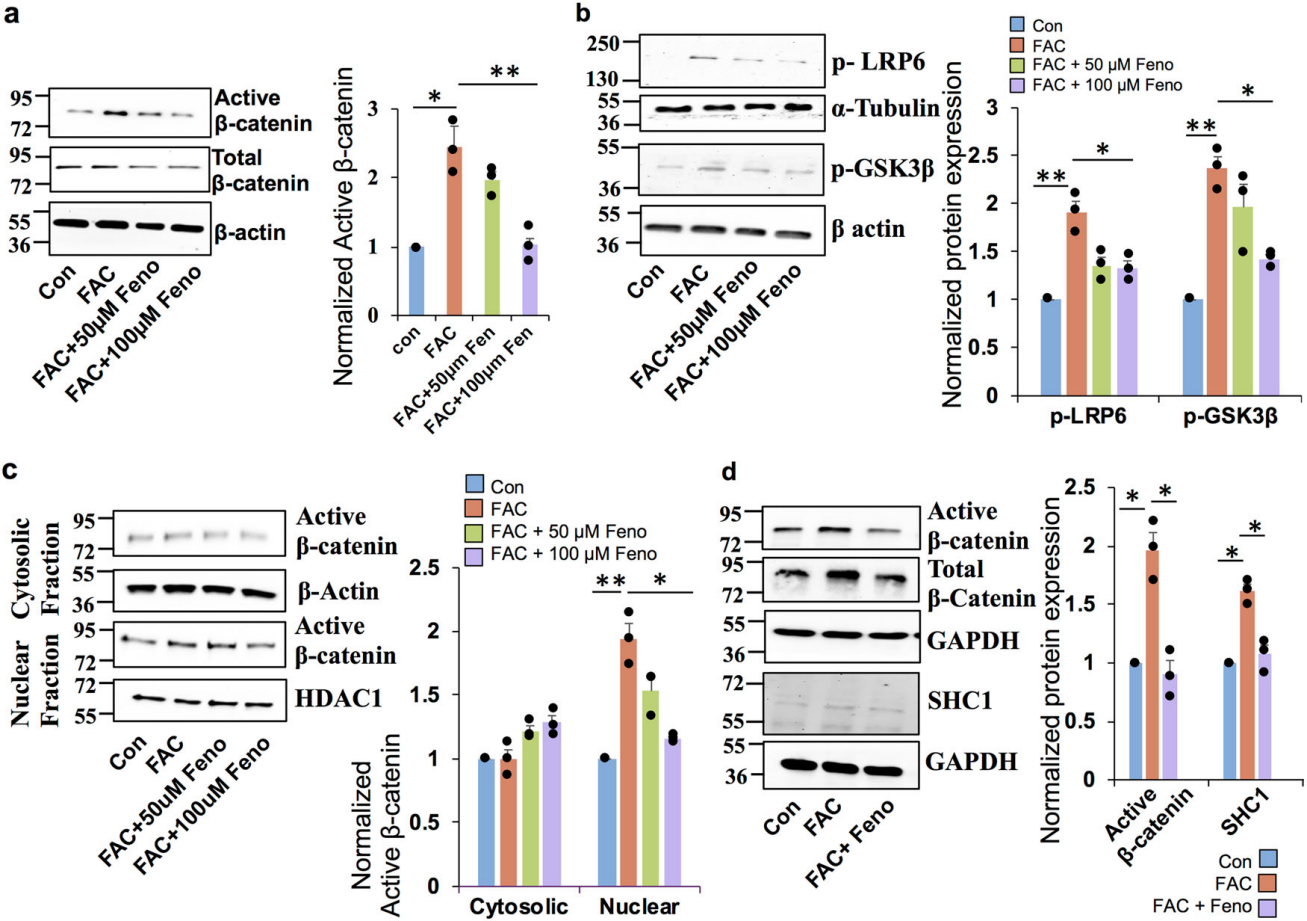
PPARα agonist fenofibrate prevents iron-induced Wnt signaling activation in ARPE19 cells. ARPE19 cells were treated with 50 and 100 µM of Fenofibrate (Feno) along with 100 µg/ml FAC for 24 h, and the protein expression of **a** active and T-β-catenin, **b** p-LRP6 and p-GSK-3β were determined by immunoblotting. β-Actin was used as a loading control. **c** Cytosolic and nuclear proteins were isolated after the cells were treated with fenofibrate in the presence of FAC for 24 h and the expression of active β-catenin was determined by western blotting. **d** Active β-catenin, total β-catenin, and SHC1 protein levels were assessed by western blotting in mouse primary RPE cells treated with FAC and fenofibrate. Blots cropped from different parts of the same gel or from different gels are separated by white space. Data presented as mean ± SE of three independent experiments. *p < 0.05; **p < 0.001.

Fenofibrate, a PPARα agonist has recently been reported to suppress Wnt3a induced β-catenin signaling in renal cells^30^. To investigate whether the iron-induced activation of canonical Wnt signaling in the retina is abrogated by fenofibrate, ARPE19 cells were treated with fenofibrate in the presence of 100 µg/mL FAC for 24 h. Treatment with fenofibrate significantly reversed the upregulation of FAC-induced active β-catenin and total β-catenin levels (Fig. 5a). Further, fenofibrate prevented the FAC-mediated upregulation of cMYC, LRP6, and p-GSK-3β (S9) expression (Fig. 5b), and significantly reduced the nuclear translocation of β-catenin in ARPE19 cells (Fig. 5c). Similarly, fenofibrate prevented the FAC-induced upregulation of active β-catenin expression significantly in the primary RPE cells (Fig. 5d). In addition, fenofibrate abrogated the FAC-mediated upregulation of p66 Shc1, an indicator of redox imbalance, in primary RPE cells (Fig. 5e). Taken together, these results indicate that FAC-induced dysregulation of canonical Wnt signaling in the retina could be prevented by fenofibrate.

### Fenofibrate is an iron chelator

**Fig. 6:**
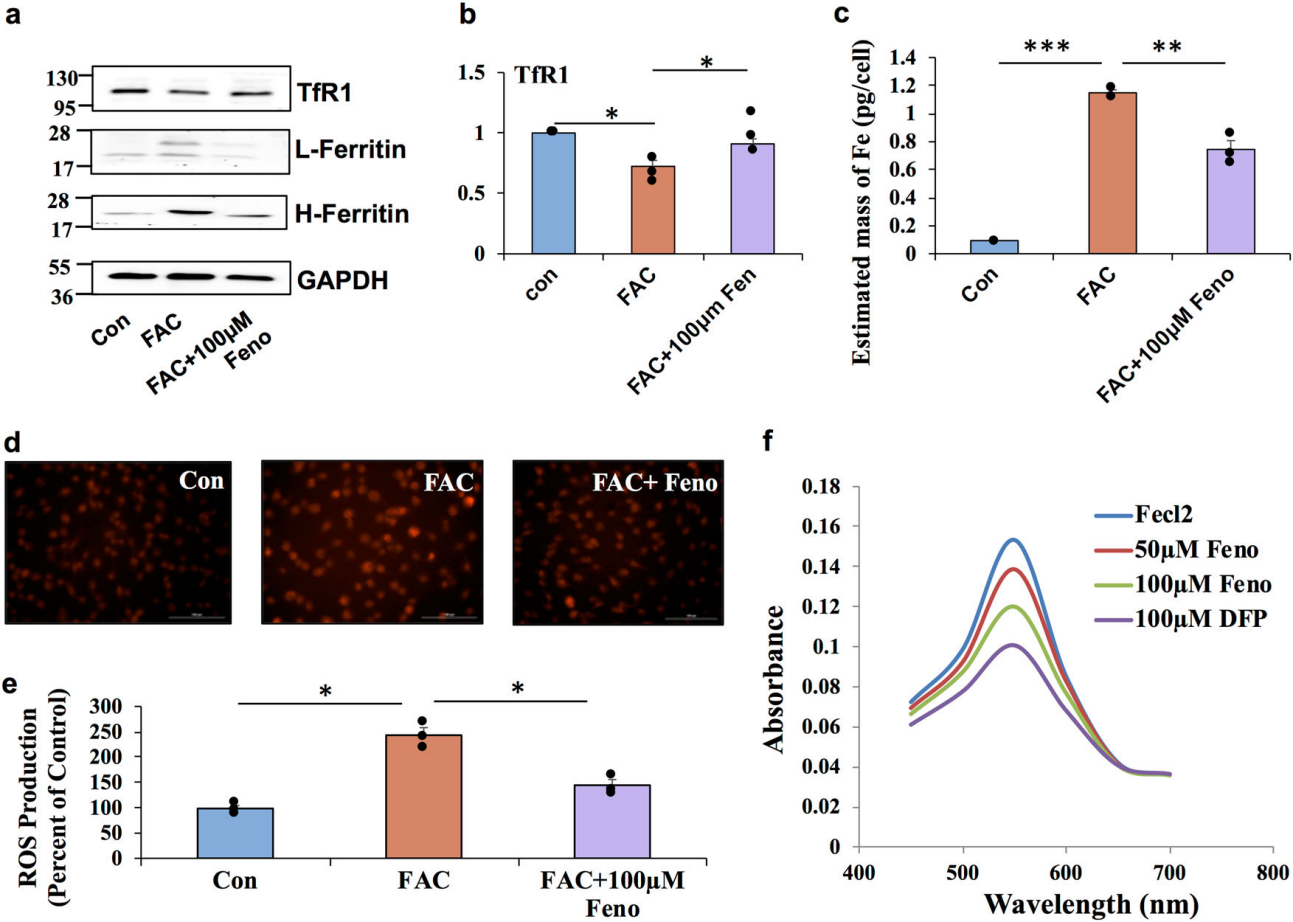
Fenofibrate prevents iron-induced Wnt signaling activation by chelating iron. **a** TfR1 and L&H ferritin protein levels in ARPE19 cells treated with FAC and fenofibrate by western blotting. **b** mRNA expression of TfR1 was determined by real-time PCR. **c** Intracellular iron content were quantified by ICP-MS. **d** Cells were stained for labile iron (Fe^2+^) using FeRhoNox-1 fluorescent imaging probe. **e** ROS generation was monitored by CM-H_2_DCFDA staining and quantified. **f** Iron-chelating activity of fenofibrate was assessed based on its ability to interfere with the formation of ferrozine-Fe2+ complex when treated along with FeCl_2_. Iron chelator deferiprone (DFP) was used as a positive control. Data presented as mean ± SE of three independent experiments. *p < 0.05; **p < 0.001, ***p < 0.0001.

The anti-cancer activity of new compounds with iron chelation capability has recently been demonstrated to be antagonizing the Wnt signaling activation^38,39^. Our experiments with DFP also indicate that iron chelation is an effective strategy to inhibit Wnt signaling. Next, we determined whether fenofibrate prevents the activation of Wnt signaling by reducing the intracellular iron levels. ARPE19 cells treated with fenofibrate normalized the expression of TfR1 and ferritin levels altered by FAC treatment (Fig. 6a), indicating that fenofibrate reduces intracellular iron accumulation. In addition, we found that fenofibrate reversed iron-mediated downregulation of TfR1 mRNA levels by reverse transcriptase PCR (RT-PCR) (Fig. 6b). Fenofibrate treatment in mouse primary RPE cells also resulted in a similar normalization of iron-mediated increase in Ferritin levels (Supplementary Fig. 2a) and decrease in TfR1 levels (Supplementary Fig. 2b). We confirmed further by direct intracellular iron estimation using inductively coupled plasma-mass spectrometry (ICP-MS) (Fig. 6c) and by FeRhonox-1 staining for labile iron in ARPE19 cells (Fig. 6d). In addition, fenofibrate treated cells showed lower ROS generation compared to the cells treated with FAC (Fig. 6e). Fenofibrate significantly inhibited the ferrozine-Fe^2+^ complex formation (Fig. 6f), indicating that fenofibrate binds to Fe^2+^ and chelates iron in turn leading to reduction in intracellular iron levels. We found that the active form of fenofibrate, fenofibric acid, also inhibited the ferrozine-Fe^2+^ complex significantly albeit with a lower potency than fenofibrate (Supplementary Fig. 3).

### Fenofibrate downregulates FAC-induced RAS signaling

**Fig. 7:**
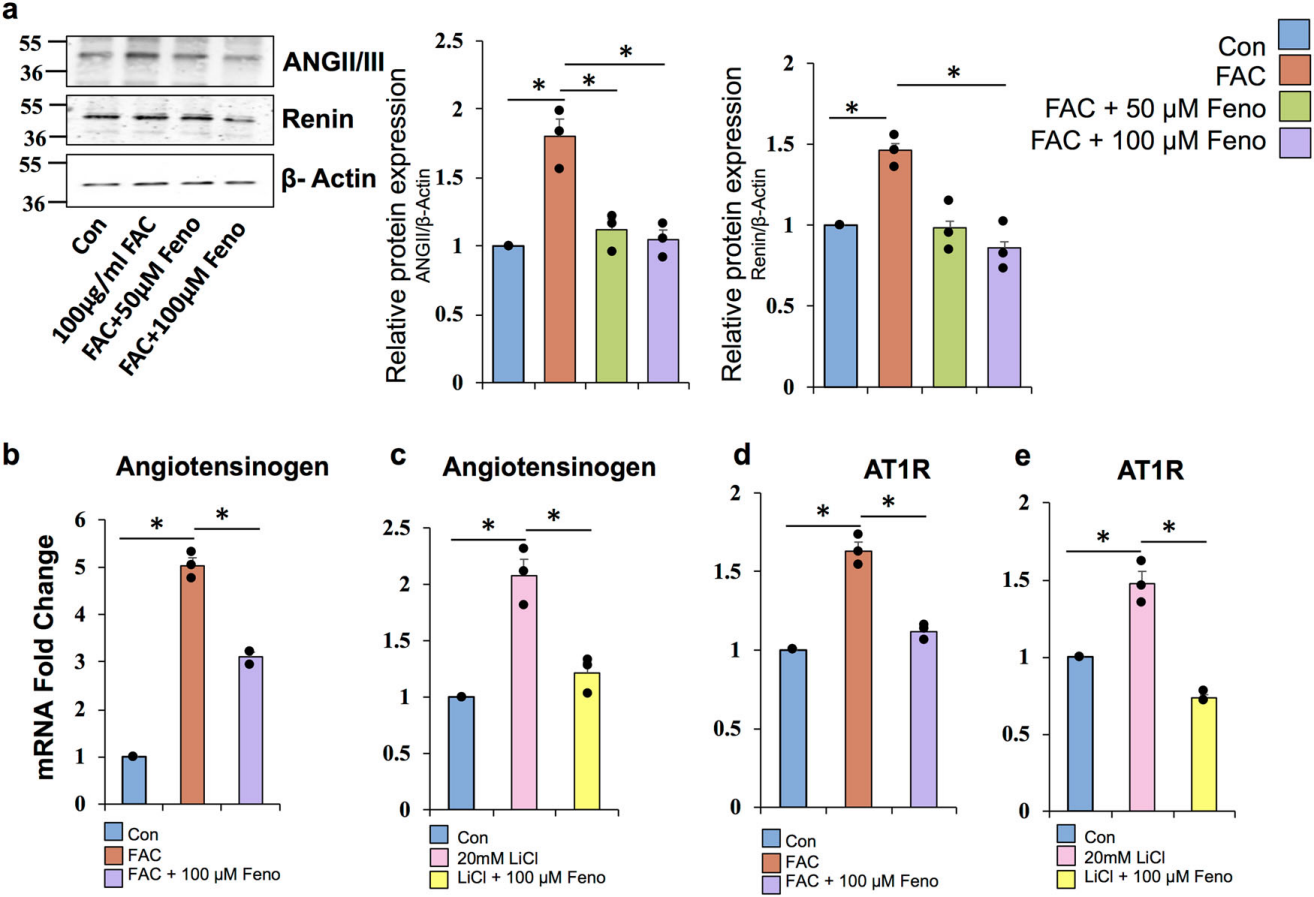
Fenofibrate downregulates FAC-induced RAS signaling. **a** Protein levels of ANG II/III and renin were quantified by western blot in RPE cells treated with fenofibrate in the presence of 100 µg/ml FAC for 24 h. **b**, **c** mRNA expression of angiotensinogen was monitored by RT-PCR. **d**, **e** mRNA expression of ATR1 was determined by RT-PCR. LiCl treatment was done as a positive control. Data presented as mean ± SE of three independent experiments. *p < 0.05.

**Fig. 8:**
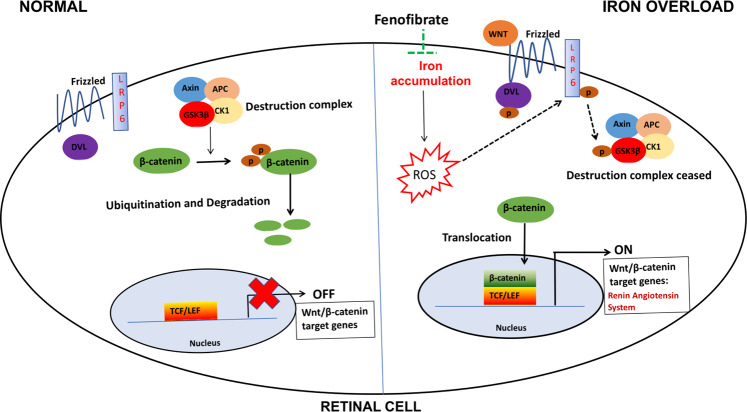
Schematic illustration. A schematic of the signaling pathways showing fenofibrate preventing iron-induced activation of oxidative stress, Wnt/β-catenin and renin–angiotensin system signaling.

Retina expresses all the components of the RAS. We investigated the effect of fenofibrate on FAC-induced RAS activation in ARPE19 cells. FAC-treated cells showed increased expression of ANG II/III and Renin, which were significantly prevented by fenofibrate treatment in RPE cells (Fig. 7a). Fenofibrate also normalized the iron and LiCl-mediated upregulation of AGT and AT1R (Fig. 7b-e), the other RAS genes that are downstream targets of Wnt signaling. These results suggest that fenofibrate prevents the RAS activation by inhibiting Wnt signaling in RPE cells as shown in Fig. 8.

## Discussion

Wnt signaling pathway is an important regulator of retinal development at various stages including retinal stem cell maintenance, ciliary body formation, cornea and lens formation, retinal field establishment, and retinal vasculogenesis^40^. Hence, mutation in many components of the Wnt signaling such as Fzd-4^41^, LRP5/6^42^, Norrin^43^, or APC^44^ result in defective retinal vasculogenesis leading to compensatory neovascularization, vascular leakage, retinopathy of prematurity, retinal detachment, or in certain cases retinal coloboma. On the contrary, aberrant activation and nuclear localization of β-catenin has been demonstrated during ocular pathologies like DR^45,46^, AMD^47–49^, proliferative retinopathy^50^, and laser induced choroidal neovascularization^51^, and blocking of Wnt signaling resulted in anti-inflammatory and anti-angiogenic effects^52–54^. There is an emerging body of evidence implicating iron accumulation in aging^4,5^ and retinal degenerative diseases^6–15^, but whether this involves Wnt signaling has not been studied. Here, we demonstrate that iron overload activates retinal canonical Wnt signaling both in vitro and in vivo. We observed that in the retina, iron inhibits GSK-3β, upregulates LRP6 and β-catenin levels, increasing the translocation of active β-catenin to the nucleus and upregulating β-catenin downstream target genes of the RAS, which are known to be regulated by TCF binding to their promoter^17^. Our results further establish that iron-mediated upregulation of Wnt/β-catenin signaling is dependent on oxidative stress as treatment with antioxidant NAC or iron chelator DFP abrogated the iron-mediated Wnt activation.

Iron overload is associated with the pathophysiology of several diseases including cancer^2^. Similarly, Wnt signaling is a major oncogenic signaling pathway underlying carcinogenesis in many tissues^55^. Under normal conditions, iron has been found to either not alter or downregulate Wnt/β-catenin signaling in colon and liver respectively^56,57^. On the other hand, our present study showing upregulation of canonical Wnt signaling by iron in the retina of WT mouse with functional APC and β-catenin indicates a strong tissue-dependent differential regulation of Wnt signaling by iron. Two independent groups have shown that multiple iron chelators inhibit Wnt/β-catenin signaling and block cancer cell growth^38,39^. In addition, lipid peroxidation product, 4-HNE treatment to RPE cells and retinal endothelial cells, has been reported to increase the levels of phosphorylated and total LRP6, β-catenin, and TCF/β-catenin downstream genes^58^. Thus, our present finding that treatment with antioxidant NAC or iron chelator DFP abrogates the iron-mediated LRP6 and Wnt activation implicates iron-catalyzed 4-hydroxenonenal as at least partly responsible for the iron-induced Wnt activation in the retina. However, future studies will be aimed at discerning the differential mechanisms involved in the tissue-dependent regulation of Wnt/β-catenin signaling by iron.

Oxidative stress is considered as one of the possible mechanisms for the progressive death of cones and rods in RP^33^. Importantly, use of antioxidants has been found to reduce rod cell death in the rd1 and rd10 mouse models of RP^33^. In addition, iron-chelating drugs delayed retinal degeneration in rd10 mouse model of RP^34^. Interestingly, a clinical case reported recently of a 35-year-old patient with a missed iron foreign body in left eye for 7 years, presenting with a pseudo-RP-like fundus^59^, indicates a causative role for iron in developing RP-like phenotype. We show that iron accumulation in rd1 mice of different ages is associated with enhanced activation of canonical Wnt signaling in the retina providing a possible mechanism by which iron regulates RP pathogenesis. Hence, iron chelation therapy as a potential preventive strategy for RP warrants further investigation. Also, how a mutation in beta subunit of the cGMP phosphodiesterase gene in rd1 mice leads to iron accumulation in the retina before cell death begins is a critical question that needs further exploration.

PPARα downregulation has been implicated in DR^29^ and AMD^60^, which may be responsible for the overactivation of Wnt signaling^30^, thereby contributing to the disease progression. PPARα activation by its agonist fenofibrate has recently been shown to attenuate several mediators of vascular damage including inflammation, endothelial dysfunction, and angiogenesis during DR and AMD^27,28,60–66^. In the present study, we found that treatment with fenofibrate prevented iron-induced dysregulation of Wnt signaling in both human and mouse RPE cells. Previous reports on small molecule drugs with iron binding and chelating activity abrogating the Wnt signaling and inhibiting the cancer cell growth^38,39^ led us to hypothesize that fenofibrate acts as an iron chelator. We found that fenofibrate reduced intracellular labile iron and ROS levels. Further, our ICP-MS and Ferrozine iron chelation assay strongly demonstrated that fenofibrate treated cells have significantly lower levels of intracellular iron by chelating it. Although we found only one clinical report on fenofibrate inducing anemia^67^, interestingly, the EFECTL Study (Effect of Fenofibrate and Ezetimibe Combination Treatment on Lipid Study), a three-arm parallel-group open-label randomized trial, reported that 52 patients undergoing fenofibrate monotherapy alone had a significant reduction in the red blood cell count and hemoglobin starting from 4 weeks after treatment was initiated until 52 weeks for the entire duration of the study^68^. Our present finding that fenofibrate is an iron chelator provides a plausible explanation for the results seen in EFECTL trial. In summary, our study demonstrates that iron accumulation in the retina inhibits GSK-3β and activates LRP6 and β-catenin signaling in an oxidative stress-dependent mechanism. In addition, we report a previously unidentified pharmacological effect of fenofibrate as an iron chelator reducing intracellular iron accumulation. This work has significant translational potential as fenofibrate could be an attractive therapeutic drug for the treatment of many chronic diseases associated with iron overload.

## Methods

### Animals

C57BL/6J mice, Axin2^LacZ/+^ mice, and C57BL/6J^*rd1/le*^ (rd1 mice) breeders were purchased from Jackson Laboratory (Bar Harbor, ME). All the mice were housed at the animal facility of Saint Louis University School of Medicine. Gender, age and weight matched animals were randomly divided into different groups as indicated in the results. All procedures involving mice were approved by the Saint Louis University Institutional Committee on Animal Use for Research and Education and were performed in accordance with the Association for Research in Vision and Ophthalmology Statement for the Use of Animals in Ophthalmic and Vision Research.

### Intravitreal and intraperitoneal injections

Considering the toxicity of FAC to the retina, holo-transferrin was injected intravitreally in mice to simulate iron overload. Briefly, animals were anesthetized with 1 : 1 mix of ketamine (8 mg/ml) and xylazine (1.2 mg/ml). Pupils were dilated with 1% atropine sulfate ophthalmic solution. Animals were injected with 1 μL of 2.4 mM holo-transferrin (Holo-Tf) or 1 μL of 2.4 mM apo-Tf as control. Twenty-four hours post injection, mice were killed and retinas were collected. For systemic iron overload, mice were administered with iron-dextran (1 g/kg body weight) intraperitoneally once a week for a duration of 8 weeks. PBS was administered intraperitoneally to the control group.

### Cell culture

The human RPE cell line, ARPE19 was procured from American Type Culture Collection (Manassas, VA) and maintained in 1 : 1 Dulbecco’s modified Eagle’s medium/F12 (DMEM/F12, Invitrogen, Gibco Corp., Grand Island, NY) supplemented with 10% fetal bovine serum (FBS) (Hyclone, Logan, UT) and 100 IU/ml penicillin and 100 μg/ml streptomycin (Invitrogen, Carlsbad, CA, USA) at 37 °C in a humidified air chamber with 5% CO_2_. For biochemical experiments, cells were seeded in a six-well plate (unless otherwise mentioned) and were utilized after reaching 90% confluence followed by a 5–6 h serum starvation before any treatment was done.

### Mouse primary RPE cell cultures

Three-week-old C57BL/6 pups were used to isolate primary RPE cells as described previously^7^. Cells were grown in 1 : 1 DMEM/F12 (Invitrogen, Gibco Corp., Grand Island, NY) supplemented with 25% FBS (Hyclone, Logan, UT) and 5% penicillin/streptomycin (Invitrogen, Carlsbad, CA, USA) at 37 °C cell culture incubator with 5% CO_2_. Purity of the cultures was verified as described previously by immunodetection of RPE65, a known marker for RPE cells ^7^.

### RNA isolation, cDNA synthesis, and RT-PCR

Cells were treated with the experimental conditions and total RNA was extracted using Trizol (Invitrogen, USA) and the quantity of the extracted RNA was assessed by measuring the absorbance at 260 nm. RNA (500 ng) was reverse transcribed to synthesize the cDNA using iScript™ cDNA Synthesis Kit (Bio-Rad, USA) according to the manufacturer’s protocol. Then, cDNA was diluted (1 : 5) and utilized to assess the gene expression by ABI Quant Studio3 real-time PCR system using iTaq™ Universal SYBR® Green Supermix (Bio-Rad, USA). The list and sequence of specific primers used are provided in Supplementary Table 1.

### Immunoblotting

Protein was extracted from the cells and the tissues using a lysis buffer (50 mM Tris, 150 mM NaCl, 10 mM EDTA) supplemented with protease inhibitors (1 μg/ml aprotinin, 1 μg/ml pepstatin, 1 μg/ml leupeptin, 1 mM phenylmethylsulfonyl fluoride, 1 μg/ml trypsin inhibitor) and 1% NP-40. The concentration of the protein was estimated by bicinchoninic acid assay (Thermo Fisher Scientific, Waltham, MA). Protein (20 µg) was subjected to SDS-polyacrylamide gel electrophoresis and transferred onto the nitrocellulose membrane. The membrane was then blocked for 1 h with 3% bovine serum albumin (fatty acid free) and incubated with the primary antibodies overnight at 4 °C. Then, the blots were washed and incubated with secondary antibodies for 90 min at room temperature (RT). Blottings were again washed and the proteins were visualized using enhanced chemiluminescence western blotting detection system (Thermo Fisher Scientific, Waltham, MA). Antibody sources and dilutions used are listed in Supplementary Table 2. All blots or gels were derived from the same experiment and were processed in parallel.

### Nuclear and cytosolic protein isolation

Cells with or without treatment were washed with PBS at 4 °C and the cytosolic and nuclear fractions of cells lysates were purified using the NE-PER™ nuclear and cytoplasmic extraction reagent (Thermo Fisher, Waltham, MA) following the manufacturer’s guidelines.

### Luciferase assay

Wnt activity was measured by using the TCF/LEF Reporter Kit (BPS Bioscience, USA). Briefly, cells were seeded at a density of 10,000 cells per well in a 96-well plate, transfected with either negative control or reporter plasmid as mentioned in the kit. Wnt pathway activity was detected using the Dual Luciferase Assay System, normalized with *Renilla*, and calculated according to the TCF/LEF Reporter Kit data sheet.

### Histological detection of labile iron

Eyecups were fixed in 4% paraformaldehyde and embedded in OCT (Optimal Cutting Temperature) compound for cryosectioning as previously described^69^. Labile iron (Fe^2+^) in retinal sections were detected using FeRhoNox-1 fluorescent imaging probe as mentioned in our recent publications^10,70^. RhoNox-1 was dissolved in dimethyl sulfoxide to a final concentration of 5 μM, added to the retinal sections, and incubated for 30 min at 37 °C in a dark chamber. The sections were then counterstained with Hoechst nuclear stain and examined by laser-scanning confocal microscopy (Carl Zeiss, Oberkochen, Germany).

### Immunofluorescence analysis

Retinal sections were fixed, blocked with 1× power block and incubated overnight at 4 °C with rabbit anti-HNE. Negative control sections were incubated without the primary antibody. Sections were rinsed and incubated for 1 h with goat anti-rabbit IgG coupled to Alexa Fluor 488. Coverslips were mounted after staining with Hoechst nuclear stain and sections were observed using laser-scanning confocal microscopy. Immunohistochemistry studies were repeated twice, and the results from these two experiments were similar.

### β-Galactosidase staining

β-Galactosidase reporter gene staining kit (Millipore Sigma, St. Louis, USA) was used to stain retinal sections from Axin2^LacZ/+^ mice according to the manufacturer’s protocol.

### Measurement of intracellular ROS production

Intracellular ROS generation was monitored by CM-H2DCFDA staining according to the manufacturer’s protocol (Molecular Probes, Thermo Fisher, Waltham, MA).

### Inductively coupled plasma-mass spectrometry

Intracellular iron levels were estimated by using ICP-MS. ARPE19 cells were cultured in T-75 tissue culture flask and treated according to the experimental conditions. At the end of the treatment, the flask was rinsed with PBS for three times and cells were detached by 0.25% trypsin-EDTA. After centrifugation, the cells were suspended in 1 ml PBS and counted. Then the cells were centrifuged at 16,100 × *g* for 10 min at 4 °C and the resultant pellet was stored at −80 °C until ICP-MS analysis. On the day of analysis, frozen cell pellets were thawed and thermally digested in 1 ml nitric acid (HNO_3_) using Mars 6 Microwave Digestion System (CEM Corporation). Digested samples were diluted for mass spectrometric analysis and total iron content of the samples was estimated using PerkinElmer NexION 2000 ICP-MS in Washington University, St. Louis. A standard calibration curve was performed prior to sample analysis. The total iron content per cell was calculated, accounting for the number of cells present, as well as dilution factors, as the mean value of three individual experiments groups.

### Iron chelation assay

The chelation of ferrous iron by fenofibrate was determined by method described previously^71^. Briefly, FeCl_2_ (50 µl, 2 mM) was mixed with different concentrations of fenofibrate. Then, 200 µl of 5 mM ferrozine solution was added to initiate the reaction followed by vigorous shaking. After incubating the reaction for 10 min at RT, the absorbance of the solution was recorded at 562 nm. The percentage inhibition of the ferrozine-Fe^2+^ complex formation was calculated as (*A*_0_ − *A*_s_/*A*_s_) × 100. Here, *A*_0_ and *A*_s_ represents the absorbance of the control and fenofibrate/fenofibric acid/DFP, respectively.

### Statistical analysis

Data are presented as mean ± SEM. The fold change for bar graphs were calculated by normalizing the sample values with the respective control values. Statistical significance was determined with the Student’s *t*-test. All the experiments were performed in triplicates. The values of *p* < 0.05 were considered significant ^48^.

### Reporting summary

Further information on research design is available in the Nature Research Reporting Summary linked to this article.

## Supplementary information

Supplementary Figures

Reporting Summary

## Data Availability

The datasets generated and analyzed during the current study are available from the corresponding author on reasonable request.
