# Supplementary material for: Fenofibrate prevents iron induced activation of canonical Wnt/β-catenin and oxidative stress signaling in the retina
Source: NPJ Aging Mech Dis. 2020 Oct 30;6:12. doi: 10.1038/s41514-020-00050-7 (PMC7599211; doi:10.1038/s41514-020-00050-7)
Supplement: Supplementary file 1 — Supplementary Figures [file 41514_2020_50_MOESM1_ESM.pdf]

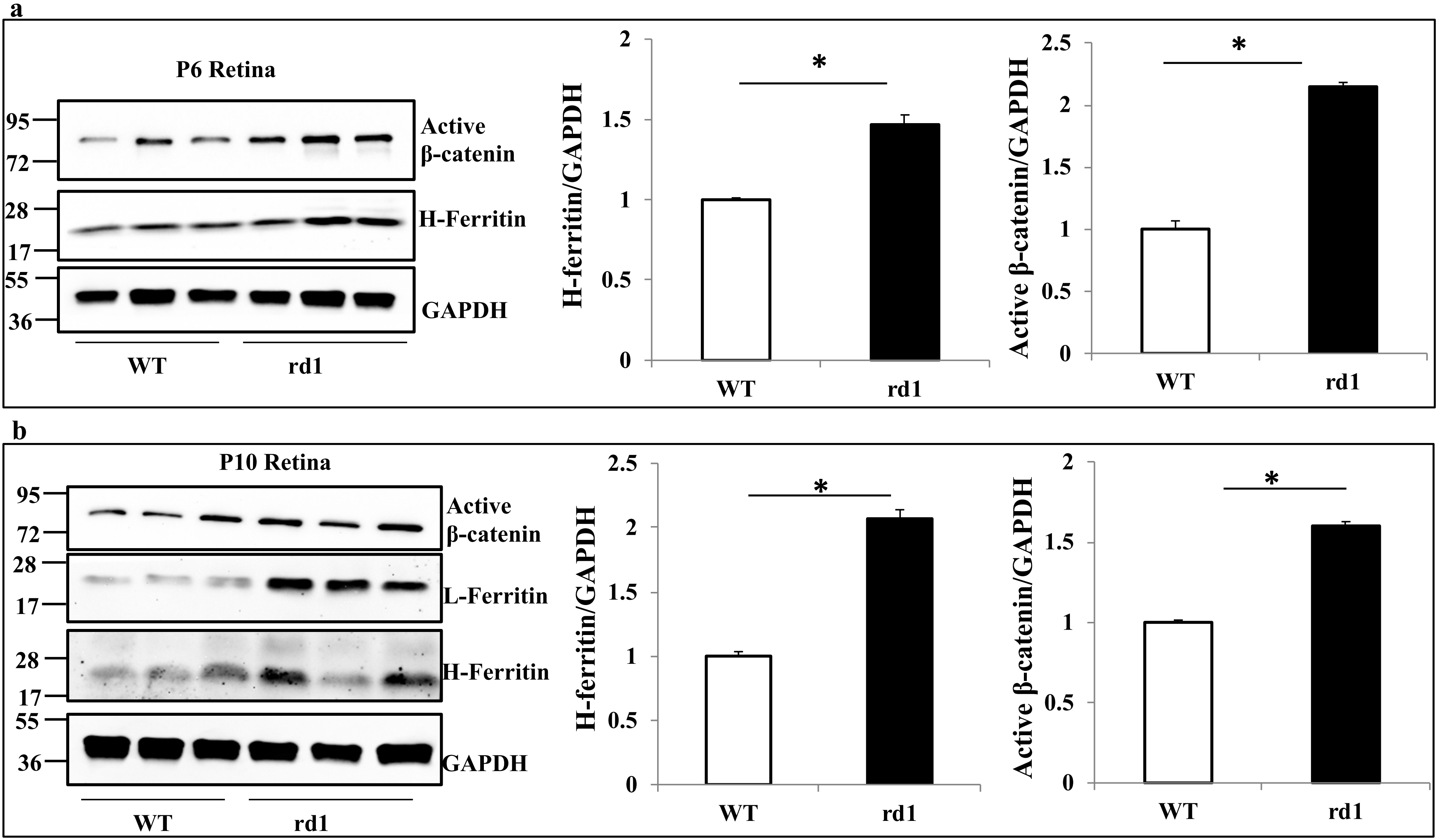

Supplementary Figure 1

Supplementary Figure 1

Heavy (H-) chain ferritin, light (L-) chain ferritin and active β-catenin protein levels were determined by immunoblotting in WT and rd1 mice at postnatal day 6 (a) and postnatal day 10 (b). Blots cropped from different parts of the same gel or from different gels are separated by white space in (a) and (b). Data presented as mean ± SE of three independent experiments; n=3 mice per group; *$p < 0.05$

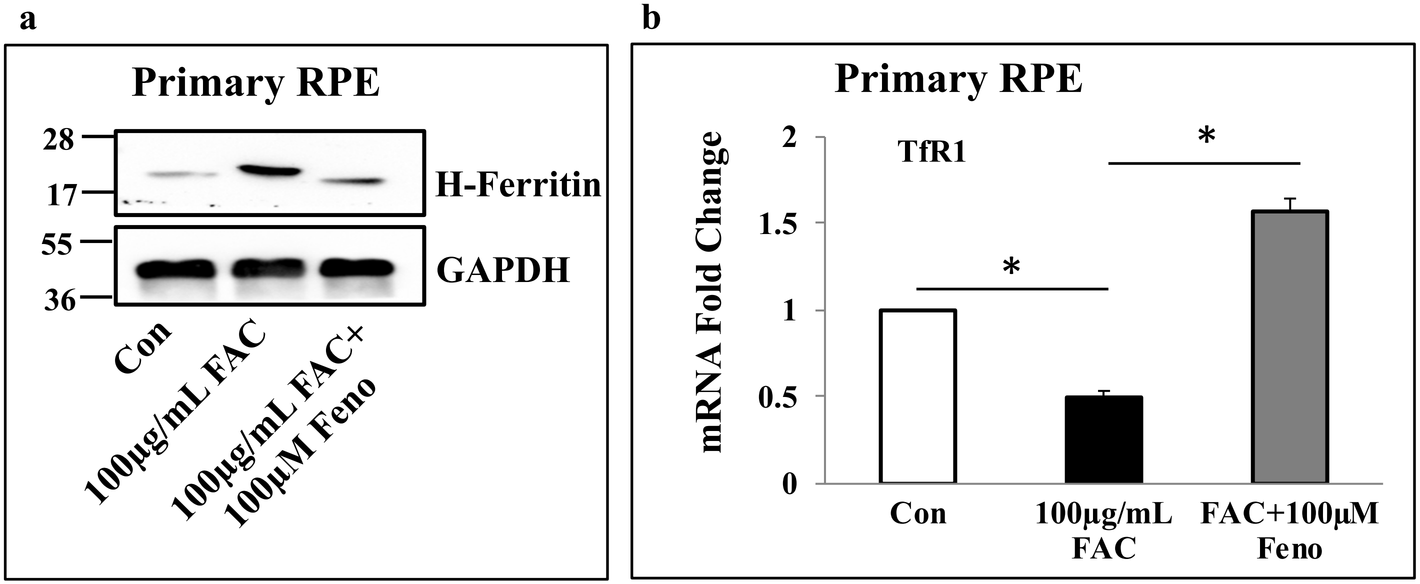

Supplementary Figure 2

Supplementary Figure 2

(a) Ferritin protein levels in mouse primary RPE cells treated with FAC and fenofibrate by western blot (b) mRNA expression of TfR1 was determined by Real-time PCR. Blots cropped from different parts of the same gel or from different gels are separated by white space in (a). Data presented as mean ± SE of three independent experiments; *$p < 0.05$

Name of the compound	% inhibition of the Ferrozine-Fe^{2+} (λmax 560)
100µM Fenofibrate	38.73684 ± 4.8
100µM Fenofibric acid	26.5501 ± 5.3
100µM Deferiprone	61.2561 ±3.9

Supplementary Figure 3

Supplementary Figure 3

Percentage inhibition of the ferrozine-Fe^{2+} complex by 100 µM fenofibrate or 100 µM fenofibric acid when treated along with $FeCl_2$. 100µM iron chelator deferiprone was used as a positive control. Data presented as mean ± SE of three independent experiments.

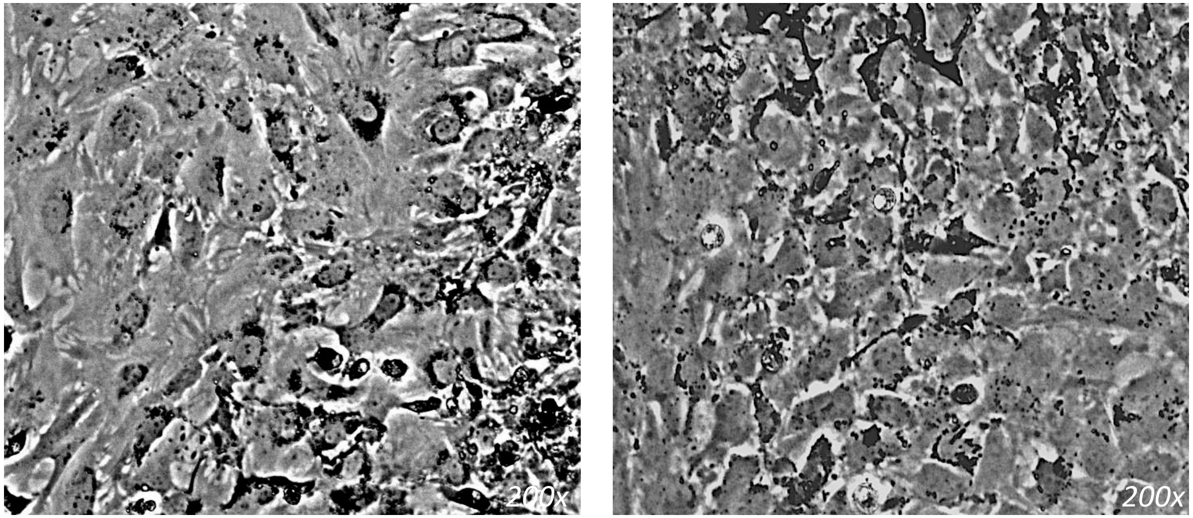

Supplementary Figure 4

Supplementary Figure 4

Representative phase-contrast images of the primary retinal pigment epithelial cells isolated from C57BL/6 mice after 6 days in culture.

Supplementary Table 1: List of primers used

h_Axin2_f	5’-CCACACCCTTCTCCAATCCA-3’
h_Axin2_r	5’-TGGACACCTGCCAGTTTCTTT-3’
h_ccnd1_f	5’-TGAGGGACGCTTTGTCTGTC-3’
h_ccnd1_r	5’-GCCTTTGGCCTCTCGATACA-3’
h_cMYC_f	5’-CCCTCCACTCGGAAGGACTA-3’
h_cMYC_r	5’-GCTGGTGCATTTTCGGTTGT-3’
h_TfR1_f	5’-GAGGACGCGCTAGTGTTCTT-3’
h_TfR1_r	5’-TGTGACATTGGCCTTTGTGTT-3’
h_HO1_f	5’-AGACACCCTAATGTGGCAGC-3’
h_HO1_r	5’-ATGGCCGTGTCAACAAGGAT-3’
m_Axin2_f	5’-GCTGCGCTTTGATAAGGTCC-3’
m_Axin2_r	5’-CGCGAACGGCTGCTTATTTT-3’
m_cMYC_f	5’-CGTTGGAAACCCCGCAGA-3’
m_cMYC_r	5’-TACGGAGTCGTAGTCGAGGT-3’
m_ccnd1_f	5’-TCAAGTGTGACCCGGACTG-3’
m_ccnd1_r	5’-CCTTGGGGTCGACGTTCTG-3’
h_18srRNA_f	5’-GTAACCCGTTGAACCCCATT-3’
h_18srRNA_f	5’-CCATCCAATCGGTAGTAGCG-3’

Supplementary Table 2: List of antibodies used

Name	Source	Cat no.	Dilution
Non-phospho (Active) β-Catenin (Ser33/37/Thr41) (D13A1) Rabbit mAb	CST	8814	1:1500
β-Catenin Antibody	CST	9562	1:1000
GSK-3β (27C10) Rabbit mAb	CST	9315	1:1000
Phospho-GSK-3-beta (Ser9) (D3A4) Rabbit mAb	CST	9322	1:1000
LRP6 (C47E12) Rabbit mAb	CST	3395	1:1000
c-Myc (D84C12) Rabbit mAb	CST	5605	1:1000
GAPDH (14C10) Rabbit mAb	CST	2118	1:5000
α-Tubulin Antibody	CST	2144	1:5000
β-Actin (8H10D10) Mouse mAb	CST	3700	1:5000
Anti-Ferritin Heavy Chain antibody	Abcam	ab65080	1:1000
Anti-Ferritin Light Chain antibody	Abcam	ab69090	1:1000
Anti-Transferrin Receptor antibody	Abcam	ab84036	1:1000
HDAC1 Antibody (10E2)	SCBT	sc-81598	1:1000
Shc Antibody (PG-797)	SCBT	sc-967	1:1000
Angiotensin II/III Antibody	Novus Biologicals	NB100-62346	1:1000
Renin Antibody	Novus Biologicals	AF4277	1:1000

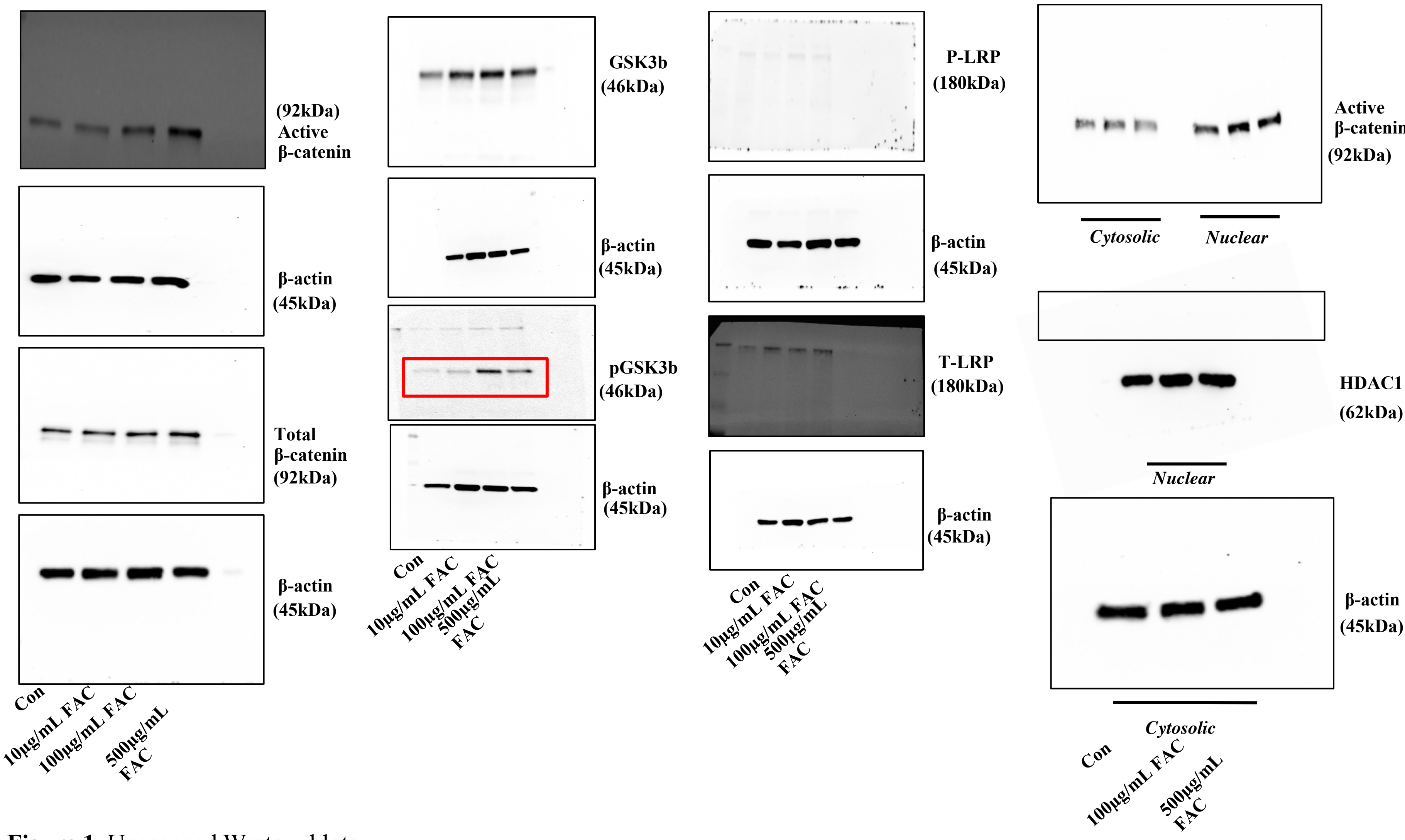

Figure 1. Uncropped Western blots

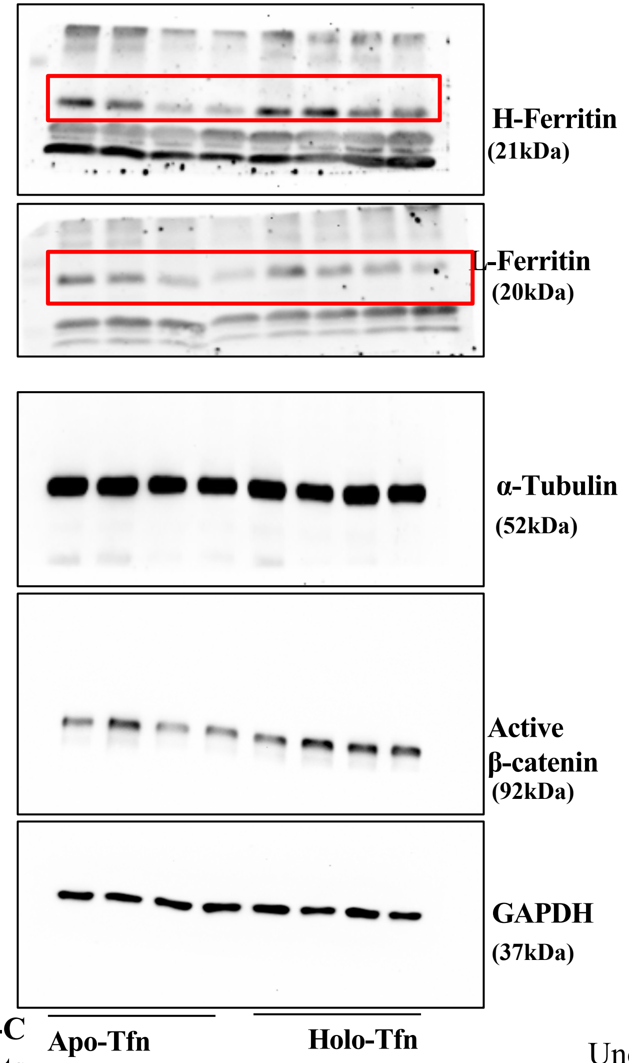

Figure 2B-C
Uncropped Western blots

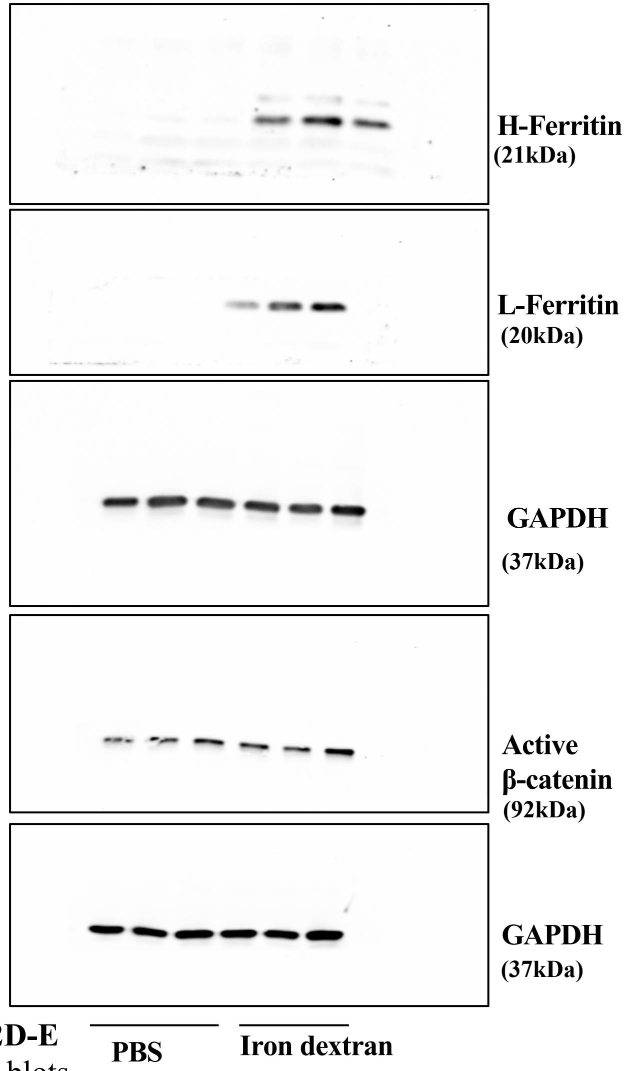

Figure 2D-E
Uncropped Western blots

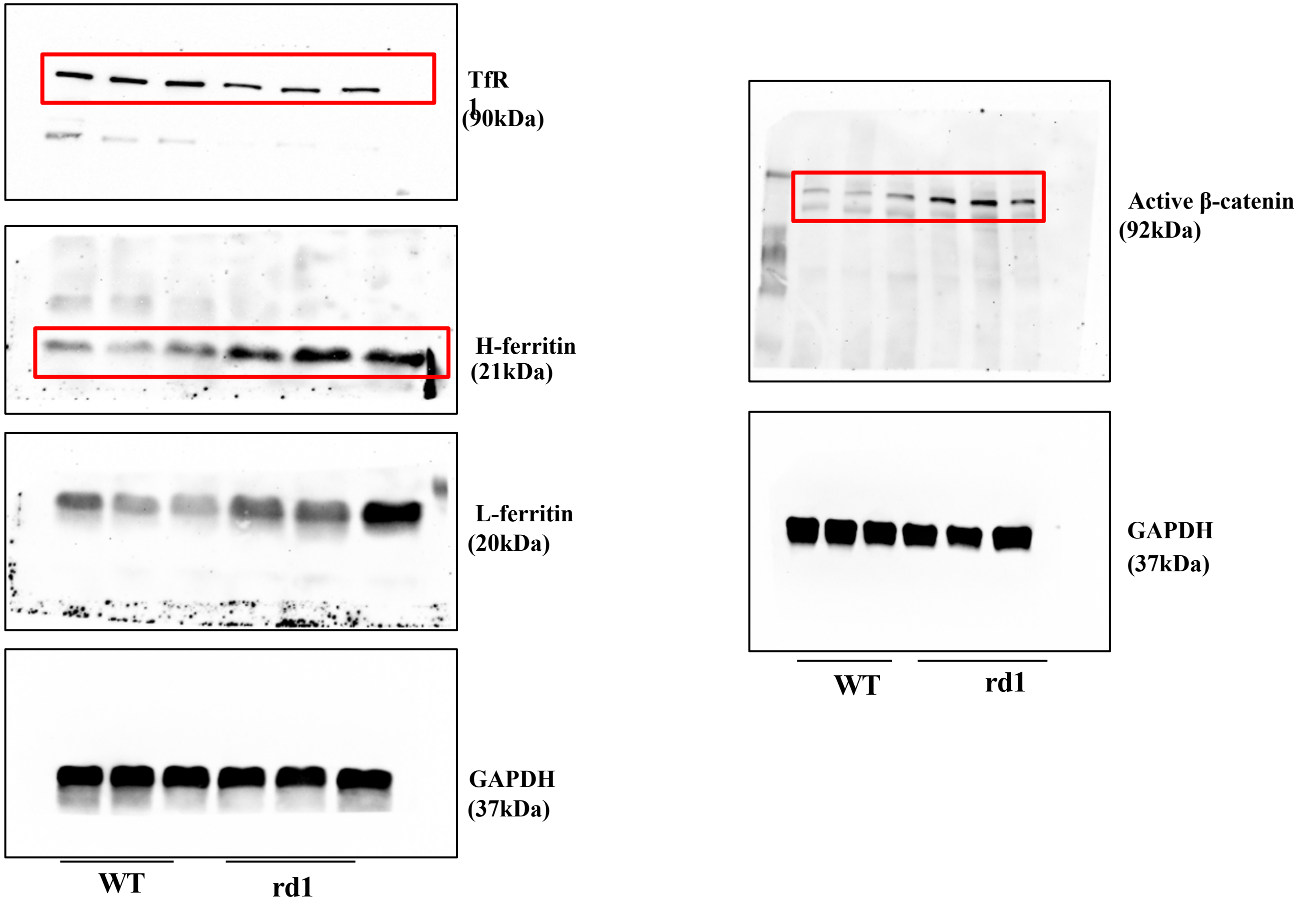

Figure 3. Uncropped Western blots

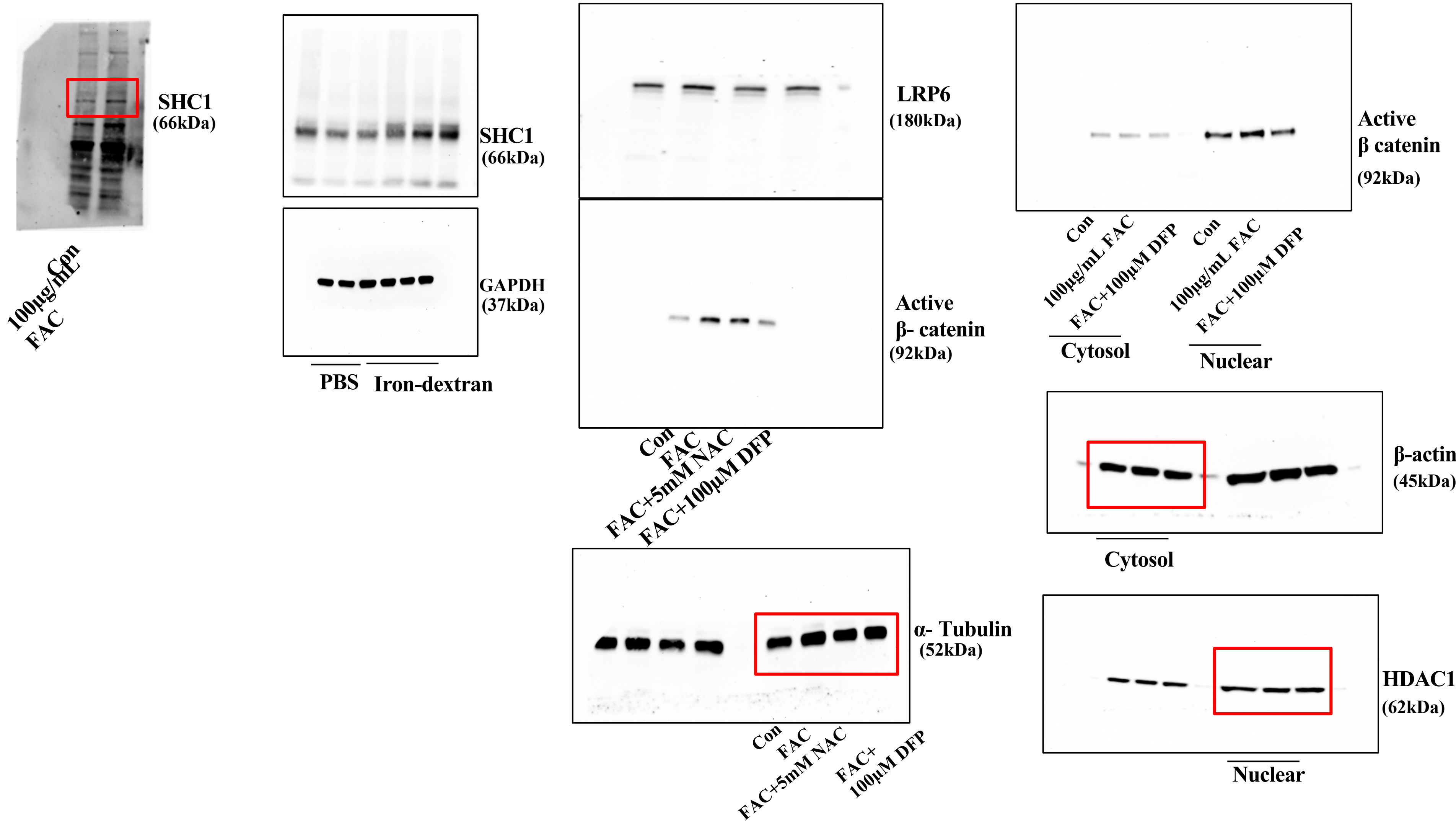

Figure 4. Uncropped Western blots

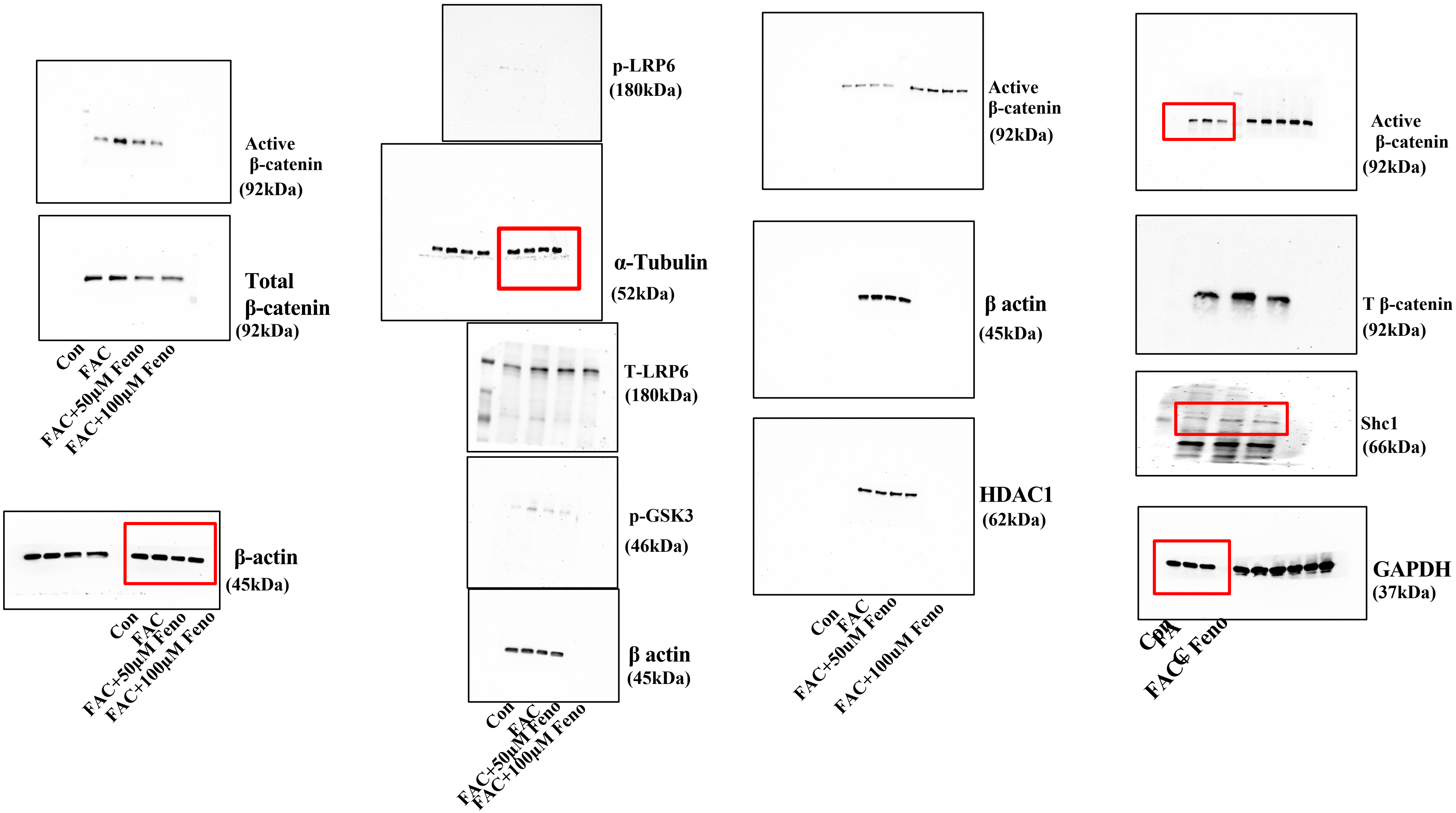

Figure 5. Uncropped Western blots

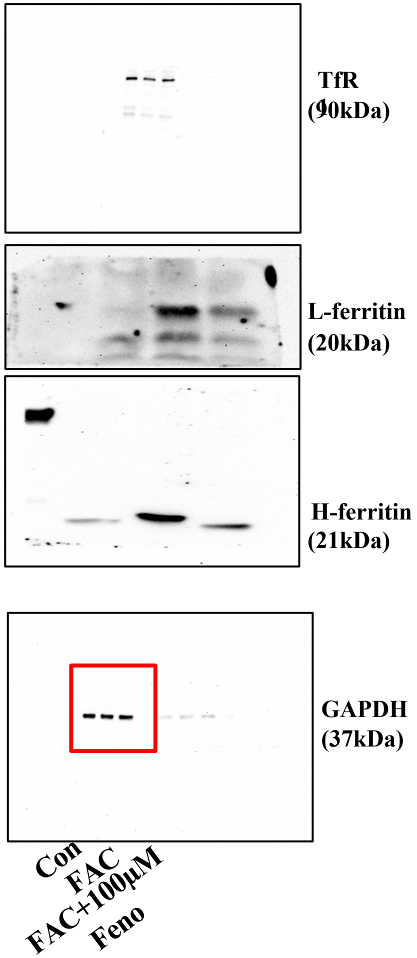

Figure 6. Uncropped Western blots

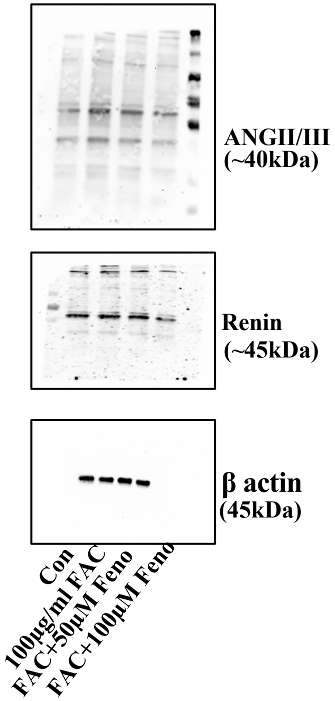

Figure 7. Uncropped Western blots
