# Supplementary material for: Fenofibrate prevents iron induced activation of canonical Wnt/β-catenin and oxidative stress signaling in the retina
Source: NPJ Aging Mech Dis. 2020 Oct 30;6:12. doi: 10.1038/s41514-020-00050-7 (PMC7599211; doi:10.1038/s41514-020-00050-7)
Supplement: Supplementary file 2 — Reporting Summary [file 41514_2020_50_MOESM2_ESM.pdf]

nature research

Corresponding author(s): Jaya P Gnana-Pakasam

Last updated by author(s): Jul 27, 2020

Reporting Summary

Nature Research wishes to improve the reproducibility of the work that we publish. This form provides structure for consistency and transparency in reporting. For further information on Nature Research policies, see our Editorial Policies and the Editorial Policy Checklist.

Statistics

For all statistical analyses, confirm that the following items are present in the figure legend, table legend, main text, or Methods section.

n/a	Confirmed	
☐	☒	The exact sample size (n) for each experimental group/condition, given as a discrete number and unit of measurement
☐	☒	A statement on whether measurements were taken from distinct samples or whether the same sample was measured repeatedly
☐	☒	The statistical test(s) used AND whether they are one- or two-sided<br>*Only common tests should be described solely by name; describe more complex techniques in the Methods section.*
☐	☒	A description of all covariates tested
☐	☒	A description of any assumptions or corrections, such as tests of normality and adjustment for multiple comparisons
☐	☒	A full description of the statistical parameters including central tendency (e.g. means) or other basic estimates (e.g. regression coefficient) AND variation (e.g. standard deviation) or associated estimates of uncertainty (e.g. confidence intervals)
☒	☐	For null hypothesis testing, the test statistic (e.g. F, t, r) with confidence intervals, effect sizes, degrees of freedom and P value noted<br>*Give P values as exact values whenever suitable.*
☒	☐	For Bayesian analysis, information on the choice of priors and Markov chain Monte Carlo settings
☒	☐	For hierarchical and complex designs, identification of the appropriate level for tests and full reporting of outcomes
☒	☐	Estimates of effect sizes (e.g. Cohen's d, Pearson's r), indicating how they were calculated

Our web collection on statistics for biologists contains articles on many of the points above.

Software and code

Policy information about availability of computer code

Data collection	QS3 software for Real time PCR, iBright imaging software for western blots, Gen5 software for Microplate reader and Zeiss software for confocal imaging
Data analysis	ImageJ software

For manuscripts utilizing custom algorithms or software that are central to the research but not yet described in published literature, software must be made available to editors and reviewers. We strongly encourage code deposition in a community repository (e.g. GitHub). See the Nature Research guidelines for submitting code & software for further information.

Data

Policy information about availability of data

All manuscripts must include a data availability statement. This statement should provide the following information, where applicable:

- Accession codes, unique identifiers, or web links for publicly available datasets
- A list of figures that have associated raw data
- A description of any restrictions on data availability

The datasets generated and analyzed during the current study are available from the corresponding author on reasonable request.

Field-specific reporting

Please select the one below that is the best fit for your research. If you are not sure, read the appropriate sections before making your selection.

☒ Life sciences ☐ Behavioural & social sciences ☐ Ecological, evolutionary & environmental sciences

For a reference copy of the document with all sections, see nature.com/documents/nr-reporting-summary-flat.pdf

Life sciences study design

All studies must disclose on these points even when the disclosure is negative.

Sample size	No statistical method was used to determine the sample size. Based on our previous published studies, the sample size used in the present study was adequately powered to detect the observed differences between the experimental groups.
Data exclusions	No data were excluded.
Replication	Replication of the experimental findings generated similar results.
Randomization	Wells in the cell culture and animals for the in vivo studies were allocated into experimental groups randomly.
Blinding	Investigators were blinded to group allocation during data collection and analysis of intracellular iron levels using inductively coupled plasma mass spectrometry.

Reporting for specific materials, systems and methods

We require information from authors about some types of materials, experimental systems and methods used in many studies. Here, indicate whether each material, system or method listed is relevant to your study. If you are not sure if a list item applies to your research, read the appropriate section before selecting a response.

Materials & experimental systems

n/a	Involved in the study	
☐	☒	Antibodies
☐	☒	Eukaryotic cell lines
☒	☐	Palaeontology and archaeology
☐	☒	Animals and other organisms
☒	☐	Human research participants
☒	☐	Clinical data
☒	☐	Dual use research of concern

Methods

n/a	Involved in the study	
☒	☐	ChIP-seq
☒	☐	Flow cytometry
☒	☐	MRI-based neuroimaging

Antibodies

Antibodies used	List of antibodies used, source and catalog numbers are mentioned under Table 2 in the Supplementary Information of the manuscript.
Validation	The antibodies were purchased commercially based on the literature citations. All the antibodies used were validated by the manufacturer. In addition, the antibodies used for western blot were validated by the presence of a band at the expected molecular weight with reproducible results.

Eukaryotic cell lines

Policy information about cell lines

Cell line source(s)	ARPE-19 cells were obtained from the American Type Culture Collection ATCC® CRL-2302™.
Authentication	The cell line used was not authenticated.
Mycoplasma contamination	The cell line was negative for mycoplasma contamination.
Commonly misidentified lines (See ICLAC register)	n/a

Animals and other organisms

Policy information about studies involving animals; ARRIVE guidelines recommended for reporting animal research

Laboratory animals	C57BL/6J, Axin2LacZ/+ and C57BL/6Jrd1/le breeders were purchased from Jackson Laboratory (Bar Harbor, ME).
Wild animals	This study did not involve wild animals.
Field-collected samples	This study did not involve field-collected samples.
Ethics oversight	All protocols involving mice were approved by the Saint Louis University Institutional Committee on Animal Use for Research and Education and were performed in accordance with the Association for Research in Vision and Ophthalmology Statement for the Use of Animals in Ophthalmic and Vision Research.

Note that full information on the approval of the study protocol must also be provided in the manuscript.
